# Health Behavior of Young People Aged 12–18 with Autism Spectrum Disorder and Intellectual Disabilities in Hungary

**DOI:** 10.3390/children12060753

**Published:** 2025-06-10

**Authors:** Ágota Barabás, Renáta J. Erdei, Mariann Móré, Viktória Pázmány, Attila Sárváry, Emil Toldy-Schedel, Anita M. Grestyák, Attila Csaba Nagy, Orsolya P. Kiss, Péter Takács

**Affiliations:** 1 Department of Health Methodology and Prevention, Faculty of Health Sciences, University of Debrecen, Sóstói u. 2–4, 4400 Nyíregyháza, Hungarypazmany.viktoria@etk.unideb.hu (V.P.); kiss.orsolya@etk.unideb.hu (O.P.K.); 2 Faculty of Health Sciences, Institute of Social and Sociological Sciences, University of Debrecen, 4400 Nyíregyháza, Hungary; 3 Department of Integrative Health Sciences, Faculty of Health Sciences, University of Debrecen, 4400 Nyíregyháza, Hungary; 4 St. Francis Hospital of Budapest, 1021 Budapest, Hungary; 5 Department of Health Informatics, Faculty of Health Sciences, University of Debrecen, 4032 Debrecen, Hungarytakacs.peter@etk.unideb.hu (P.T.); 6 Department of Health Informatics, Faculty of Health Sciences, University of Debrecen, 4400 Nyíregyháza, Hungary

**Keywords:** autism spectrum disorder, intellectual disability, health, health behavior

## Abstract

Introduction: People with disabilities are characterized by suboptimal health and lower self-rating health. Their need for health care is greater, they often have a higher prevalence of health problems and they have more difficulty accessing health care. The aim of this study was to assess the health behaviors and health indicators of 12–18-year-old young people with intellectual disabilities and autism spectrum disorder, and to explore their school-related perceptions in the Northern Great Plain region of Hungary. Materials and Methods: A cross-sectional questionnaire survey was conducted with the participation of 185 young people. A custom questionnaire was used, based on the Health Behavior in School-aged Children (HBSC) survey, assessing eating habits, oral care, physical activity, mental well-being, and self-reported health status. The sample was categorized into three groups: the ID1 (Intellectual Disability level 1) group, encompassing young individuals with mild intellectual disability; the ID2 group, encompassing young people with moderate intellectual disability; and the ID+ASD group, encompassing young individuals affected by both intellectual disability and autism spectrum disorder. Results: Consumption of various food types was below optimal levels. Low intake of fruits and vegetables was common, with only 21.6% of the respondents consuming fruit daily and 23.8% consuming vegetables daily. ID1 group reported significantly higher rates of nervousness several times a week (17.8% vs. 5.6% and 6.9%, *p* < 0.001), sleep difficulties (28.8% vs. 7.4% and 15.5%, *p* = 0.032), and dizziness (9.6% vs. 1.9% and 3.4%, *p* = 0.022) compared to the other two groups. A third school-related factor, related to negative emotions, showed a near-significant difference (*p* = 0.064), suggesting that students with both autism spectrum disorder and intellectual disability perceive lower levels of acceptance from teachers. On school-free days, computer usage was significantly highest in the ID+ASD group; 50% of them used a computer for at least 4 h per day. Conclusions: To improve mental well-being among affected children, psychological support and the implementation of mental health programs are recommended. In addition to teaching stress management techniques and coping mechanisms, integrating relaxation techniques into comprehensive developmental programs—both individually and in groups—is advised. For teachers, it is recommended to acquire disability-specific communication strategies.

## 1. Introduction

The health status of the Hungarian population is unfavorable. Health status indicators such as life expectancy at birth, premature mortality, and mortality are the worst in Hungary compared to EU Member States [1]. Individuals in lower socioeconomic status are at increased risk of morbidity and mortality, have lower psychosocial capacity, and are more likely to engage in health risk behaviors. In adolescence, it is particularly important to pay attention to the impact of social determinants on health, as this is the period in life when unhealthy habits are established, risky behaviors are more frequent and peer relationships are more likely to be reordered, which is also the case in disability. The association between disability and disadvantage is well known [2,3].

Previous studies have investigated adolescents’ health behavior in Hungary, of which the following are the major ones.

HBSC research is a collaborative, international study with WHO, which takes place every four years. Hungary has been participating in the study since 1985. More than 50 countries in Europe and beyond are participating in the research, which examines the health behavior of school-age young people. The results of the 2021/2022 data collection showed that students typically consume low amounts of fruit and vegetables. In Hungary, less than 30% of young people consume fruit and vegetables on a daily basis. Just over a fifth of them get enough physical activity, and more than a fifth are overweight or obese. Young people spend most of their free time sitting down. The prevalence of psychosomatic symptoms is high, with more than half of children experiencing at least two psychosomatic symptoms on a weekly basis [4].

Another important survey on health risk behaviors is the European School Survey on Alcohol and other Drugs (ESPAD), which measures the health risk behaviors of 15–16-year-olds in 35 countries, in which Hungary has been participating since 1995. According to the results of the 2024 survey, 59% of students reported good mental well-being. Hungary is one of the countries with the lowest mental well-being. Smoking among young people is on the rise, with the highest rate in Hungary (51%). The use of computers and online games is also on the rise [5].

People with disabilities are characterized by suboptimal health and a lower self-rating of health. Their need for health care is greater, they often have a greater prevalence of health problems and they have more difficulty in accessing health care. They are three times more likely to suffer from diabetes, three times more likely to be malnourished, and three times more likely to be denied access to health care [6,7]. Young people with disabilities, particularly those with autism spectrum disorders and intellectual disabilities, are more likely to be overweight and obese than children without disabilities. Adolescents with disabilities have also been reported to be at higher risk of developing cardiovascular disease [7,8,9].

Health status of children with autism spectrum disorder (ASD): children with ASD are 1.5 times more likely to be overweight or obese, and in cases of severe ASD, this risk is three times higher than in cases of mild ASD [10]. Atypical cognitive, social, motor, and behavioral difficulties hinder interaction with peers, and these limitations also reduce children’s physical activity. As a result, a sedentary lifestyle negatively affects their health and further isolates them from their peers [11,12].

Children with ASD are more frequently affected by comorbid conditions such as otitis media (middle ear infections), and they tend to have higher rates of antibiotic use [13]. Nutritional problems are also more common, including insufficient calcium and protein intake. Food selectivity contributes to the development of poor dietary habits [14].

Health status of children with intellectual disabilities: They are more likely to be affected by obesity and diabetes and have a higher incidence of developmental disorders, cardiovascular and respiratory diseases, mental health problems, and a shorter life expectancy. Adults with intellectual disabilities tend to be less physically active than the general population [14,15]. They also experience oral health problems at a higher rate, such as tooth decay, gingivitis, and edentulism. In addition to the higher prevalence of these diseases, more severe occurrences are also common. Poor oral hygiene has been linked to cardiovascular diseases, diabetes, and respiratory illnesses [16].

Adolescents with disabilities generally have poorer overall health than the average population, which justifies the need to monitor their health status and health behaviors. Comorbidities often associated with autism spectrum disorders and intellectual disabilities can further worsen health status.

Autism spectrum disorder (ASD) is a comprehensive developmental disorder that involves lifelong impairment in cognitive development of the nervous system. Its core characteristics include abnormal or impaired development in social interaction and communication, as well as a narrow range of interests and repetitive behaviors. The manifestations of the disorder can vary greatly depending on the individual’s developmental level and chronological age [17,18].

Intellectual disability typically involves a pervasive impairment of the nervous system, resulting in globally delayed psychomotor development across all functional domains. The categories of intellectual disability are as follows: mild: IQ 52–69; moderate: IQ 36–51; severe: IQ 20–35; and profound: IQ 19 or below [19,20,21].

Multiple disabilities are defined as the presence of at least two distinct impairments—either identical or different—that are not directly causally related to each other [19].

The goal of the research is to assess the health behaviors, health indicators, and school perceptions of 12–18-year-old young people with intellectual disabilities and autism spectrum disorder in the Northern Great Plain region of Hungary. The following questions will be examined: which areas of health behavior of young people with intellectual disability and autism are unfavorable and require intervention? Which disability group is most vulnerable in the area of school acceptance? Is there a correlation between the severity of intellectual disability and parameters indicating mental well-being?

## 2. Materials and Methods

### 2.1. Sampling

The study was conducted in 12 randomly selected institutions from the special schools of three counties in the Northern Great Plain region. Special schools refer to institutions with special education, where the class size, teacher qualifications, and curriculum differ from mainstream education [22]. These schools use a curriculum that incorporates injury-specific methodologies during special education. The sample selection was comprehensive in the sense that every young person meeting the inclusion criteria was surveyed. The inclusion criteria were as follows: 12–18 years of age, affected by intellectual disabilities or autism spectrum disorder, residing in the Northern Great Plain region, and voluntarily agreeing to participate in the survey. The exclusion criterion was the presence of severe intellectual disabilities.

### 2.2. Data Collection

As the first step, the research team obtained permission for the study from the principals/directors of the special schools. Special schools refer to institutions with specialized education, where the class size, teacher qualifications, and curriculum differ from mainstream education [23]. Following the institutional approval, the research team sent an informational letter and a consent form to the children and their parents. Out of the students from the selected schools, 189 agreed to participate in the survey. During the survey, 188 paper-based questionnaires were filled out. Interviewers were present during the completion of the questionnaires to ensure respondents’ understanding. The presence of interviewers was required in most cases during the data collection, and children required assistance in many cases. During the data cleaning process, three additional questionnaires were excluded due to failure to meet the data collection requirements (more than 50% incomplete responses, presumably unserious or improper completion). The final sample size consisted of 185 valid questionnaires (see Table 1). The data collection took place between September 2021 and June 2022.

At the beginning of data processing, based on the research objectives, the sample were categorized into three groups: the ID1 (Intellectual Disability level 1) group, encompassing young individuals with mild intellectual disability; the ID2 (Intellectual Disability level 2) group, encompassing young people with moderate intellectual disability; and the ID+ASD group, encompassing young individuals affected by both intellectual disability and autism spectrum disorder. The third study group is the ID+ASD group: young people with intellectual disability 1, 2 and autism spectrum disorder (ID+ASD), which includes young people with both ID and ASD. As autism spectrum disorder is a specific symptom complex, it was considered necessary to separate the students affected from their peers with intellectual disability alone. The symptom cluster of autism spectrum disorder may affect the outcome of the research differently in several areas. In the case of young people with intellectual disability on the spectrum, we therefore anticipated different results on a number of points.

The group classifications followed internationally recognized standards. The *International Classification of Names of Diseases (BNO)* is used as a basis for the disability groups, which can be clearly used to differentiate intellectual disability. For the third group, autism spectrum disorder was the primary criterion, as this symptom complex distinguishes people with autism from those with intellectual disability alone.

### 2.3. Measurement

The questionnaire was designed to examine and contextualize patterns of health behavior and to collect data on mental well-being and social environment. Own questionnaire was based on the internationally validated Health Behavior in School-aged Children (HBSC) survey questionnaire, assessing eating habits, oral care, physical activity, mental well-being, and self-reported health status [4].

It contains a total of 55 questions. Weight and height are based on self-report. A short response was requested to indicate place of residence, possible developmental occupation.

In addition, the data collection included the following blocks: (a) demographic information: age, gender, parental education, place of residence, and self-reported financial situation, body image; (b) health behavior and lifestyle: brushing teeth, smoking habits, and alcohol consumption; (c) assessment of perception of school; d) leisure activities.

### 2.4. Statistical Analysis

The main sociodemographic characteristics of the sample were explored through percentage distributions. The interrelations of the four examined question groups (psychosomatic behaviors, eating habits, school-related perceptions, leisure activities) were analyzed according to the three disability groups using the χ^2^ test (independence test) and the related exact test (Fisher’s test; Monte Carlo estimation—the *p*-value of the chi-square test is estimated through simulation). Exploratory Factor Analysis (EFA) was used to aggregate the content of the question groups. The factor variables derived from the analysis were also examined according to the three disability groups (ANOVA and Tamhane’s T2 post-hoc test, and Kruskal–Wallis non-parametric test for confirmation of results). Multinomial logistic regression analysis was conducted for the complex examination of the factor variables and disability groups.

Data analysis: In the first step of the analysis, sociodemographic percentages were calculated, followed by the examination of the relationship between the variables in cross-tabulation analyses. In the case of low cell frequencies, Monte Carlo estimation was performed. Results were presented in APA format. Additional parameters were examined using factor analysis to reveal factors that cannot be directly measured but which determine the behavior of persons with disabilities in the background.

The suitability of the method was confirmed by the Kmo and Bartlett tests, where KMO values and Bartlett test values were used to interpret the validity of the factor model. Multinomial logistic regression was then used to examine the relationship between disability groups and the new factor model variables. At the end of the results chapter, the statistical methods used in the study are presented in a table.

The significance level used in the tests was 0.05. Data analysis was conducted using the SPSS (v24) software package.

### 2.5. Ethical Approval

The completion of the questionnaire was anonymous, and the individuals participating in the research could not be identified. The research team carried out their work in compliance with the applicable laws, professional guidelines, and recommended ethical codes. The data collection and processing complied with the relevant regulations. The research was approved by the Medical Research Council, Scientific and Research Ethics Committee (https://ett.okfo.gov.hu/tukeb/ (accessed on 6 July 2021), approval number: IV/5706-1/2021/EKU).

## 3. Results

The results of the analysis are presented in this chapter in the following order and structure: first, the sociodemographic characteristics of the sample are briefly described, followed by the calculations for the main question groups of the questionnaire. Each section summarizes in a table the results of the correlation analysis for the three disability groups (ID1, ID2, ID+ASD), followed by the results of the Exploratory Factor Analysis (EFA) for the question group. The chapter concludes by examining the behavior of the new variables generated by the factor analysis from the perspective of the three disability groups; finally, each factor is incorporated into a multinomial logistic regression to analyse the three groups.

### 3.1. Sociodemographic Characteristics

Boys were over-represented in the survey, with 64.9% of young people completing the questionnaire being boys and 35.1% girls. Nearly a third of respondents (28.1%) were aged 18, with those aged 12–13 making up 22.2% of the sample. The 14–15 age group accounts for 25.9% of respondents and the 16–17 age group for 23.8%. Additionally, 39.5% of the young people in the sample have mild intellectual disability (ID1), 29.1% have moderate intellectual disability (ID2), and 29.1% have intellectual disability plus autism spectrum disorder (cumulative disability) (ID+ASD). More than 80% of the children are in a family, 8.1% of the respondents live in a boarding school, 2.7% in a children’s home, and 8.6% in foster care (Table 1).

### 3.2. Analysis of a Set of Questions on Psychosomatic Behavior

A correlation exists between the majority of psychosomatic symptoms and type of disability. The prevalence of headaches did not differ between the three groups—the presence of headaches and group membership were independent. Respondents with mild intellectual disability (ID1) had a significantly greater proportion of anxiety (17.8% vs. 5.6%; 6.9% *p* < 0.001), sleep difficulties (28.8% vs. 7.4%, 15.5% *p* = 0.032), and dizziness (9.6% vs. 1.9%, 3.4% *p* = 0.022) compared to the other two groups. A significantly lower proportion of young people with moderate intellectual disability (ID2) were found to have irritability and moodiness several times a week. One third of young people with both intellectual disability and autism (ID+ASD), 13.0% of students with moderate intellectual disability (ID2), and 26.0% of students with mild intellectual disability (ID1) reported about tiredness several times a week (Table 2).

The exploratory factor analysis (EFA) of psychosomatic symptoms yielded the following findings: seven of the original nine variables related to psychosomatic symptoms indicating mental well-being were retained after multiple runs, as two could not be assigned to the same factor. After examining the variables “sleep-related difficulties” and “abdominal pain”, these were finally removed from the model. The cumulative percentage increased from 53.30% to 63.73% after exclusion. In a factor analysis of the parameters indicating psychosomatic symptoms (KMO = 0.833, *p* < 0.001), those indicating emotional life and mental state appeared to be related for the respondents (PSY factor 1), with physical symptoms forming a separate group (PSY factor 2) (Table 3).

### 3.3. Analysis of the Set of Questions on Dietary Habits

The two distinct parts of the set of questions are the set of questions on the consumption of alcoholic beverages and the set of questions on dietary habits—this is presented at the beginning of this section, followed by the results of the exploratory factor analysis of the set of questions.

Dietary habits: The consumption of different food types is suboptimal. The consumption of fruit and vegetables is low, with 21.6% of young people consuming fruit and 23.8% consuming vegetables per day. Dairy products are consumed daily by slightly more than a third of students (34.1%). 45.9% of students never eat liver. Fish is also not a preferred food, with 48.1% of respondents never eating it and 4.3% eating it several times a week.

Alcohol consumption habits: In terms of alcohol consumption, the majority of students never drink alcoholic beverages. However, some respondents drink alcohol at least once a week. Weekly consumption of alcoholic beverages is highest among those with mild intellectual disability (ID1), except for sweet liqueurs. Weekly consumption of sweet liqueurs is highest in ID2 (3.7%). A significantly (*p* = 0.003) higher proportion of young people in ID1 (15.1% vs. 1.9%, 12.3%) consume short drinks (Table 4).

Young people with mild intellectual disability had the highest rate of daily brushing (*p* = 0.039). A total of 12.1% of students with intellectual disability and autism spectrum disorder, 9.6% of students with mild intellectual disability and 7.4% of students with moderate intellectual disability rarely or never brush their teeth.

The results of the exploratory factor analysis (EFA) of nutritional habits: 23 variables related to diet and alcohol consumption were combined into an initial factor model. After reviewing the results, the classification of nine variables was uncertain (they were relatively equally weighted in several factors, making their classification ambiguous). These variables were: tea without sugar, tap water, syrup, carbonated soft drinks, wholegrain cereals, refined cereals, milk and milk products, energy drinks, and sweets. These nine variables were eventually removed from the model, after individually examining their role in the latter. The final model captures 71.67% of the total content of the 14 variables included (the initial 23 variables covered 61.43% of the content with seven variables). The results of the factor analysis (KMO = 0.752, Bartlett’s test *p* = 0.00) show the types of food and beverages that students think are related. According to this, alcoholic beverages (NUT factor 1) form a well-defined group, fruit and vegetables, which are part of the healthy food category, also form a separate category (NUT factor 2), soft drinks, crisps (NUT factor 3), and fruit juices and sugared tea (NUT factor 4) are perceived as cohesive by the respondents. Similarly, liver and sea fish are separate composite variables (NUT factor 5) (Table 5).

### 3.4. Analysis of a Set of Questions on Perceptions of School

The majority of young people like their school. There is no significant correlation between the groups, but it can be observed that young people with learning difficulties like their school the least (Table 6).

The results of the exploratory factor analysis (EFA) of questions about school perceptions unveiled the following: the nine variables on perceptions of school were combined into a continuous factor model. After a review of the results, the classification of one variable was uncertain (“my classmates are eligible”) and was present in other factors with similar weight, thus making its classification unclear. This variable was finally removed from the model after a more detailed analysis of the model. The eight variables included in the final model (KMO = 0.687, *p* = 0.00) capture 59.241% of the total content in three factors (54.919% of the initial nine variables were also covered by three factors). The variables are grouped by positive and negative emotions, except for one factor. Thus, there are two well-defined groups and one factor with a separate variable: positive feelings from teachers and classmates (SCH factor 1); negative attitudes from teachers and students (SCH factor 2); and positive teacher acceptance (teachers accepting students as they are), which does not fall into either category and appears as a separate factor among students (SCH factor 3) (Table 7).

### 3.5. Analysis of a Set of Questions on Leisure Activities

Computer use on school days is reduced for all three groups. Computer use outside the school day is highest for young people with intellectual disability plus autism spectrum disorder (ID+ASD); 50% use computers for at least 4 h per day. Young people in the ID2 group use computers for the least amount of time both in and out of school (Table 8).

The results of the exploratory factor analysis (EFA) of questions about spending free time: the 12 variables on leisure activities were pooled in an initial factor model. After reviewing the results, the classification of one variable was uncertain (the variable appeared in other factors with a non-significant weigth, thus making its classification unclear). The variable “bored” was finally dropped from the model after examination. The model captures 50.21% of the total content of the 11 variables included. The result of the factor analysis (KMO = 0.671, *p* = 0.00) shows that four categories are separated. These categories are as follows: art, sports, talking to friends, going on a trip, going on a tour, reading (SFT factor 1); talking on the phone, playing music, listening to music, helping parents (SFT factor 2); TV and computer use (SFT factor 3); and drama class (SFT factor 4) (Table 9).

### 3.6. Behavior of Factor Variables by Disability Group

To explore possible differences between the three established disability groups (ID1, ID2, ID+ASD), we used the ANOVA test, the complementary Tamhane T2 post-hoc test, and a non-parametric Kruskal–Wallis test. The behavior of the group averages was examined for each of the 14 factors. Of the factors in the mental health questionnaire, the first factor (PSY 1—emotional state questions) showed a significant difference (*p* = 0.000). The first factor, mental well-being/psychosomatic symptoms, which includes variables related to emotional state, showed a significant difference (*p* = 0.00) between the ID1 and ID2 groups and between ID+ASD and ID2 (*p* = 0.00).

- From the group of questions on nutrition and alcohol consumption, the first factor (NUT 1—questions on alcohol consumption) showed a significant difference (*p* = 0.003). The results showed that the ID1 group was significantly different (*p* = 0.045) from the ID2 and ID+ASD groups in terms of alcohol consumption.
- In the school perception question group, the third factor (SCH 3—teacher acceptance) showed a significant difference (0.002). Factor 3 on school, which includes student acceptance by the teacher, was significantly different between ID1 and ID2 groups (*p* = 0.03), and there was also a difference between ID1 and ID+ASD (*p* = 0.065).
- The first factor for leisure time (SFT 1—sports, talking to friends, hiking, art) showed a significant difference (*p* = 0.000).For the first factor for leisure time, which includes “ideal” leisure activities, there is a significant difference (*p* = 0.00) between ID1 and ID2 groups and between ID1 and ID+ASD (*p* = 0.00).

One of the conditions of the ANOVA procedure is the normality of the variables. To test this, the one-sample Kolmogorov–Smirnov test was used. The results showed that only one variable exhibited normality (NUT Factor 3 (soft drinks and chips), *p* = 0.200). Although the number of items in the sample (N = 185) suggests that ANOVA is robust to minor violations of normality, a non-parametric Kruskal–Wallis test was also performed as a confirmatory analysis to check and confirm the results of the ANOVA tests.

According to the Kruskal–Wallis test, the following is true:

- Factor 1 (PSY 1), indicating mental health, which is related to positive emotions, is significant (*p* = 0.000).
- The first factor of diet and alcohol consumption (NUT 1), which measures alcohol consumption, is significant (*p* = 0.037).
- School-related factor 3 (SCH 3), which is related to negative emotions, shows a near-significant difference (*p* = 0.064).
- There is a significant difference for the first factor (SFT 1) (*p* = 0.000) and the fourth factor (SFT 4—the drama circle) (*p* = 0.009).

Accordingly, a total of five factors showed significant differences between the groups compared to the four factors in the ANOVA analysis.

### 3.7. Multinomial Logistic Regression Model of Factor Variables to Examine Disability Groups

Verification of the results from other perspectives can be conducted using multinomial logistic regression. This method can be used to identify in more detail the factors that distinguish one disability group from another. A multinomial regression predicting/adjusting the probability of belonging to the three groups (ID1, ID2, ID+ASD) induced a significant model (χ^2^ (28) = 73.98, *p* < 0.001). The model fit was found to be adequate (Deviance: χ^2^ (336) = 324.63, *p* = 0.662); Nagelkerke’s pseudo R^2^ was 0.375 (medium explanatory power). The results showed that three of the included factors showed significant differences between the groups (Table 10).

- Mental health factor 1 (PSY 1—the presence of emotional state, e.g., nervousness, irritability) showed a significant difference between the moderate intellectual disability (ID2) and mild intellectual disability (ID1) groups (B = 0.88, SE = 0.27, *p* = 0.001, OR = 2.42, 95% CI [1.42–4.12]).
- The alcohol-related factor (NUT 1) in the MLR model did not show significant differences between groups, as previously reported.
- School-related Factor 3 (SCH 3 “My teachers accept me as I am”) showed a negative correlation for the ID+ASD group (B = −0.54, SE = 0.21, *p* = 0.011, OR = 0.58, 95% CI [0.38–0.88]). This could be explained by the different behavioral pattern of autism spectrum disorder.
- For the first factor of entertainment (SFT 1—“ideal” leisure time), a difference between the ID2 and ID1 groups (B = 0.75, SE = 0.25, *p* = 0.03, OR = 2.12, 95% CI [1.29–3.49]) and between the ID2 and ID+ASD groups (B = 0.85, SE = 0.25, *p* = 0.001, OR = 2.34, 95% CI [1.44–3.78]) was found. This suggests a more active leisure time for people with moderate intellectual disability (ID2).
- Several bivariate interactions were included in the model tests (e.g., leisure time x mental health, leisure time x school perception, mental health x school perception), but none of them showed significant effects, suggesting that the variables have independent effects between groups.

### 3.8. Summary of Models

Table 11 summarizes the results of the studies, which largely confirm each other. Differences between groups were found in two cases: NUT 1 (consumption of alcoholic beverages) and SFT 4 (leisure activities). The difference in SFT 4 can be statistically explained by the very low frequency of drama club activity—four individuals reported this activity. The difference observed in alcohol consumption is almost significant in the MLR model (between the ID1 and ID2 groups, *p* = 0.091) (Table 11).

## 4. Discussion

The percentage of somatic symptoms related to mental well-being, such as headaches and stomach aches, affect more than 40% of young people with moderate intellectual disability. Psychological symptoms indicating negative emotional well-being, such as depression and nervousness, are significantly higher among young people with moderate intellectual disability and students with autism spectrum disorder in addition to intellectual disability, compared to students with mild intellectual disability. Sleep difficulties and irritability are the most common psychological symptoms among young people with disabilities. Overall, we find an unfavorable prevalence of parameters indicating mental well-being. Our findings are in line with those of Stadnick and colleagues (2022) in a study of young people with ASD, which also highlighted mental health problems among young people with ASD [21].

Since 2012, the Hungarian Comprehensive School Health Promotion Program has been a requirement for all public schools in Hungary, focusing on mental health promotion alongside healthy eating and physical activity, with the aim of ensuring that all children benefit from programs that effectively improve their health and promote their overall physical, mental, and emotional well-being [24]. A shortcoming of the program is that it is not adapted for disabilities, including intellectual disability and autism spectrum disorder.

The prevalence of unfavorable mental parameters is also a problem at the international level. According to the HBSC 2022 survey data, the most common mental symptom is fatigue and the most common physical symptom is headache [4].

Screen time of more than 4 h a day is a feature of some young people with disabilities, which can also increase mental vulnerability [25].

Dietary habits are poor and do not comply with the current “Smart Plate” recommendation, which calls for several servings of fruit and vegetables a day, something less than a third of young people with disabilities do [25]. International figures are not much lower, with slightly more than a third of adolescents meeting the HBSC [15]. The frequency of tooth brushing is also below optimal levels, with students with multiple disabilities brushing their teeth the least. One of the main barriers to dental treatment is access to services [16]. Results from a study by Vermair and colleagues (2021) showed that adolescents with intellectual disabilities have poorer oral hygiene compared to their non-disabled peers [26]. The majority of the population we studied had never consumed alcohol, but it is striking that some of them consume some form of alcohol on a weekly basis.

Being on the autism spectrum does not necessarily entail a higher level of emotional strain in the same way it does for students with intellectual disabilities. Individuals affected by intellectual disability and autism spectrum disorder behave differently not only based on the type of disability but also on its severity. The specific needs associated with autism spectrum disorder, such as deficits in social and interpersonal relationships, atypical behaviors, non-standard forms of communication and situations requiring flexibility, make it difficult to interact and communicate with children. In our study, young people with both intellectual disability and autism spectrum disorder perceived lower levels of teacher acceptance, confirming the need to improve communication. This raises the question: what causes the emotional difficulties of youth with intellectual disabilities? Could it be linked to the increasingly common phenomenon of bullying? Among this population, bullying is more likely to be sensed instinctively rather than consciously recognized, which could explain the lack or complete absence of effective coping strategies in their behavioral repertoire. The factor encompassing negative emotional/mental states showed no significant difference between students with mild intellectual disabilities and those affected by both intellectual disabilities and autism spectrum disorder. However, differences were observed based on the severity of the disability: young people with intellectual disabilities are more frequently exposed to negative emotions compared to students with learning disabilities.

A higher rate of active leisure activities was observed in the ID+ASD group compared to the ID1 group, which may suggest that social relationships and physical activity are influenced not only by abilities but also by environmental support and equal access. The ‘Active Leisure Activities’ (SFT 1) factor indicates higher activity levels amongst youth with moderate intellectual disabilities, likely due to closer parental supervision.

Screen time among youth with disabilities is generally unfavorable, with students on the autism spectrum showing significantly the highest levels of screen use—a finding also supported by earlier research [15]. Symptoms reflecting emotional states related to mental health (PSY 1 factor) correlate with the severity of the disability; higher levels of intellectual disability are associated with worse emotional strain. The ‘School Perception’ (SCH 3) factor shows that students with both intellectual disability and autism spectrum disorder experience lower levels of acceptance from teachers than ID1 and ID2 groups.

Strength and limitations: The strength of the study is that it surveyed a previously less studied group using a validated questionnaire on a relatively large sample. Limitation may include difficulties related to intellectual disability and autism spectrum disorder, such as interpreting texts, processing information in different ways. We recommend developing a digital questionnaire in the future that applies the disability-specific methods used in the research, such as visual aids, pictograms, and the principles of easy-to-understand communication. More specifically, it is important to use simple language in the questionnaire and to repeat key words. Use pictures and symbols. Train interviewers who can help children fill out the questionnaires with positive reinforcement.

## 5. Conclusions

Based on the results, several directions for intervention have emerged. Negative emotional states related to mental well-being require urgent intervention, especially among young people with intellectual disabilities. Specific interventions are also important for young people on the autism spectrum, as the ways they experience psychological stress can vary. It is recommended to establish a field of health development tailored to special needs, covering not only physical and mental health but also social health. This would require the development of adapted methods that combine approaches from health promotion, mental hygiene, and special education.

For young people with disabilities, differentiated health development proves to be effective and can lead to long-term results [27]. Psychological symptoms require psychological support, and learning stress management techniques and coping mechanisms can help manage this, alongside developing injury-specific mental health support programs. The structuring of leisure time, offline friendships, and the promotion of sport as a leisure activity can also positively affect physical and mental health, which, in turn, can reduce risk behaviors [28,29]. Mental health screening of adolescents with disabilities is recommended as a priority for primary health care [21]. However, we need to keep in mind the role of socioeconomic background in the development of health behavior [30].

When designing interventions, it is important to consider the differences between types and severity levels of disabilities. As such, developing the mental well-being and resilience of students with more severe intellectual disabilities should be a priority. The lower perception of teacher acceptance highlights the need for increased communication support with pupils with intellectual disabilities and autism spectrum disorder. Teachers are generally more accepting of students with mild intellectual disabilities and less accepting of students on the autism spectrum [31]. This experience was confirmed in our study; however, since the perception of acceptance depends on the child’s subjective experience, it is likely that, in many cases, it is not the teacher’s actual rejection but rather the student’s difficulty in understanding the communication. It is key that teachers learn disability-specific communication strategies such as easy-to-understand communication or the Picture Exchange Communication System (PECS) methodology.PECS is an alternative communication method that provides students with a functional communication system using a multidisciplinary approach. The methodology is based on the use of pictograms [32]. The use of easy-to-understand communication ensures the clear transmission of information by minimizing communication noise, even through the use of pictures and illustrations [33].

It is recommended to develop an inclusive culture within educational communities in two directions: on one hand, for teachers, and on the other, for peers—through discussions and educational support specifically for teachers [34]. In addition, organizing experiential marketplaces involving parents, professionals, and teachers can be beneficial, as a supportive community and safety net surrounding students can effectively function as a complex system promoting overall well-being.

## Figures and Tables

**Table 1:** Sociodemographic characteristics of the sample (N = 185).

Characteristics		N (%)
Gender	Boy	120 (64.9)
	Girl	65 (35.1)
Age	12–13 years old	41 (22.2)
	14–15 years old	48 (25.9)
	16–17 years old	44 (23.8)
	18 years old	52 (28.1)
Intellectual disability	ID1—mild (student with learning disability)	73 (39.5)
	ID2—moderate (intellectual disability)	54 (29.1)
Autism spectrum disorder plus intellectual disability	ID+ASD—mild/moderate	58 (31.4)
Child’s place of residence	At home with your family	149 (80.5)
	In college	15 (8.1)
	In a children’s home	5 (2.7)
	In foster care	16 (8.69)

**Table 2:** Prevalence of psychosomatic symptoms by disability type.

%					
Charasteristic		Type of Disability			
		ID1	ID2	ID+ASD	Total
Headache	Seldom or never	57.5	51.9	65.5	58.4
	At least once a month	27.4	42.6	22.4	30.3
	Several times a week	15.1	5.6	12.1	11.4
N.S.					
Stomachache	Seldom or never	43.8	53.7	69.0	54.6
	At least once a month	38.4	40.7	24.1	34.6
	Several times a week	17.8	5.6	6.9	10.8
χ^2^ (df = 4, N = 185) = 11.979, *p* = 0.018; Monte-Carlo sample *p* = 0.020, 95% CI [0.017–0.024]					
Back pain	Seldom or never	74.0	79.6	69.0	74.1
	At least once a month	16.4	14.8	25.9	18.9
	Several times a week	9.6	5.6	5.2	7.0
N.S.					
Sadness/depression	Seldom or never	41.1	46.3	31.0	39.5
	At least once a month	26.0	48.1	39.7	36.8
	Several times a week	32.9	5.6	29.3	23.8
χ^2^ (df = 4, N = 185) = 3.758 *p* = 0.440; Monte Carlo sample *p* = 0.479, 95% CI [0.466–0.492]					
Nervousness	Seldom or never	31.5	61.1	27.6	38.9
	At least once a month	23.3	33.3	37.9	30.8
	Several times a week	45.2	5.6	34.5	30.3
χ^2^ (df = 4, N = 185) = 28.801, *p* = 0.000; Monte Carlo sample *p* = 0.000, 95% CI [0.000–0.000]					
Difficulty sleeping	Seldom or never	47.9	59.3	60.3	55.1
	At least once a month	23.3	33.3	24.1	26.5
	Several times a week	28.8	7.4	15.5	18.4
χ^2^ (df = 4, N = 185) = 10.577, *p* = 0.032; Monte Carlo sample *p* = 0.031, 95% CI [0.026–0.035]					
Dizziness	Seldom or never	69.9	90.7	87.9	81.6
	At least once a month	20.5	7.4	8.6	13.0
	Several times a week	9.6	1.9	3.4	5.4
χ^2^ (df = 4, N = 185) = 11.471, *p* = 0.003; Monte Carlo sample *p* = 0.029, 95% CI [0.024–0.033]					
Irritability	Seldom or never	50.7	55.6	32.8	46.5
	At least once a month	20.5	37.0	37.9	30.8
	Several times a week	28.8	7.4	29.3	22.7
χ^2^ (df = 4, N = 185) = 15.575, *p* = 0.003; Monte Carlo sample *p* = 0.001, 95% CI [0.000–0.002]					
Tiredness	Seldom or never	46.6	48.1	25.9	40.5
	At least once a month	27.4	38.9	43.1	35.7
	Several times a week	26.0	13.0	31.0	23.8
χ^2^ (df = 4, N = 185) = 11.060, *p* = 0.026; Monte Carlo sample *p* = 0.019, 95% CI [0.015–0.022]					

**Table 3:** Rotated component matrix of psychosomatic symptoms.

	Component	
	PSY 1.	PSY 2.
He/she was nervous	0.825	0.237
He/she was irritable	0.818	0.208
He/she was bad tempered	0.796	0.283
He/she was tired	0.774	0.100
He/she had back pains	0.105	0.793
He/she had headache	0.294	0.700
He/she felt dizzy	0.167	0.668

Note: the first column contains the variables included, the following columns illustrate the factors and factor weights obtained after the procedure.

**Table 4:** Frequency of alcohol consumption by disability.

%					
Characteristics		ID1	ID2	ID+ASD	Total
Wine or wine compote, champagne	Never	71.2	88.9	80.7	79.3
	Monthly or less often	23.3	11.1	17.5	17.9
	Once a week at least	5.5	0.0	1.8	2.7
N.S.					
Other alcoholic drinks	Never	79.5	90.7	87.7	85.3
	Monthly or less often	17.8	9.3	10.5	13.0
	Once a week at least	2.7	0.0	1.8	1.6
N.S.					
Short drinks	Never	79.5	98.1	86.0	87.0
	Monthly or less often	15.1	1.9	12.3	10.3
	Once a week at least	5.5	0.0	1.8	2.7
χ^2^ (df = 4, N = 184) = 10.525 *p* = 0.032; Monte Carlo sample *p* = 0.014, 95% CI [0.011–0.017]					
Alcoholic soft drinks	Never	80.8	90.7	82.5	84.2
	Monthly or less often	13.7	7.4	17.5	13.0
	Once a week at least	5.5	1.9	0.0	2.7
N.S.					
Beer	Never	79.5	88.9	82.5	83.2
	Monthly or less often	17.8	11.1	15.8	15.2
	Once a week at least	2.7	0.0	1.8	1.6
N.S.					
Sweet liqueur	Never	83.6	92.6	91.2	88.6
	Monthly or less often	13.7	3.7	8.8	9.2
	Once a week at least	2.7	3.7	0.0	2.2
N.S.					

**Table 5:** Rotated component matrix of nutritional habits.

	Component				
	NUT 1.	NUT 2.	NUT 3.	NUT 4.	NUT 5.
Wine or coke with wine, champagne	0.891				
Other alcoholic beverages	0.877				
Shot of drink	0.855				
Alcoholic soft drinks	0.837			−0.122	0.108
Beer	0.815				−0.113
Sweet liqueur	0.771	−0.132			0.121
Vegetables		0.879			
Fruit		0.852		−0.106	
Coke or other soft drink			0.862		−0.153
Chips			0.839	−0.114	0.231
Fruit juice				0.788	
Tea with sugar				0.776	
Liver					0.837
Sea fish		0.384		0.127	0.671

Note: the first column contains the variables included; the following columns show the factors and factor loadings obtained after the procedure. Blank cells contain factor weights less than 0.1 and are, therefore, not shown.

**Table 6:** Perception of school (N = 184).

%					
		ID1	ID2	ID+ASD	Total
Feelings about school	Does not like	19.2	15.1	6.9	14.1
	likes	80.8	84.9	93.1	85.9
N.S.					

**Table 7:** Rotated component matrix.

	Component		
	SCH 1.	SCH 2.	SCH 3.
Your teachers care about your personality	0.704	0.110	
Most of your classmates are helpful	0.678		
Their teachers treat them fairly	0.670		0.275
Her classmates like to be with her	0.669		
He trusts his teachers very much	0.654		−0.327
Feel not accepted by your classmates	−0.138	0.798	0.224
Feel not accepted by your teachers	0.136	0.774	−0.271
Your teachers accept you as you are	0.100		0.919

Note: the first column contains the variables included; the following columns show the factors and factor loadings obtained after the procedure. Blank cells contain factor weights less than 0.1 and are, therefore, not shown.

**Table 8:** Computer usage by disability.

%					
		ID1	ID2	ID+ASD	Total
On school days	None or less than one hour	39.7	46.3	27.6	37.8
	2–3 h	31.5	33.3	36.2	33.5
	4 h or more	28.8	20.4	36.2	28.6
N.S.					
On school days	None or less than one hour	30.6	33.3	15.5	26.6
	2–3 h	29.2	42.6	34.5	34.8
	4 h or more	40.3	24.1	50.0	38.6
χ^2^ (df = 4, N = 184) = 10.578, *p* = 0.032; Monte Carlo sample *p* = 0.027, 95% CI [0.023–0.031]					

**Table 9:** Rotated component matrix of leisure activities.

	Component			
	SFT 1.	SFT 2.	SFT 3.	SFT 4.
Attending art classes	0.739		−0.117	
Taking part in sport	0.641	0.204	0.238	−0.210
Talking to friends	0.583	0.157	−0.104	
Hiking	0.570		0.397	0.172
Reading	0.421	0.136	0.346	0.332
Using the phone		0.654		−0.118
Playing music or listening to music		0.626		0.189
Helping their parents	0.183	0.614		0.225
Watching TV or videos		−0.138	0.806	0.150
Using the computer, playing video games		0.438	0.605	−0.294
Attending drama class		0.158		0.834

Note: the first column contains the variables included; the following columns show the factors and factor loadings obtained after the procedure. Blank cells contain factor weights less than 0.1 and are, therefore, not shown.

**Table 10:** Multinominal logistic regression likelihood ratio tests.

Effect	Model-Fitting Criteria	Likelihood Ratio Tests		
	−2 Log Likelihood of Reduced Model	Chi-Square	df	Sig.
Intercept	330.346	5.713	2	0.057
**PSY 1**	347.427	22.794	2	**0.000**
PSY 2	328.345	3.712	2	0.156
NUT 1	328.476	3.843	2	0.146
NUT 2	325.426	0.793	2	0.673
NUT 3	324.640	0.007	2	0.996
NUT 4	326.614	1.981	2	0.371
NUT 5	326.299	1.666	2	0.435
SCH 1	327.761	3.128	2	0.209
SCH 2	326.751	2.117	2	0.347
**SCH 3**	332.580	7.947	2	**0.019**
**SFT 1**	344.021	19.388	2	**0.000**
SFT 2	326.743	2.110	2	0.348
**SFT 3**	330.158	5.525	2	**0.063**
SFT 4	325.335	0.702	2	0.704

Significant values are highlighted in bold.

**Table 11:** Summary of significant differences in factors for ID1, ID2, and ID+ASD disability groups.

Method	Significant Factors			
	Mental health question group	Nutrition and alcohol question group	School perception question group	Leisure activities question group
Anova	PSY 1	NUT 1	SCH 3	SFT 1
Kruskal–Wallis	PSY 1	NUT 1	SCH3	SFT 1, SFT 4
MLR	PSY 1	-	SCH3	SFT 1

## Data Availability

The raw data supporting the conclusions of this article will be made available by the authors on request.
